# Alterations to the urinary metabolome following semi-controlled short exposures to ultrafine particles at a major airport

**DOI:** 10.1016/j.ijheh.2021.113803

**Published:** 2021-08

**Authors:** Liza Selley, Ariana Lammers, Adrien Le Guennec, Milad Pirhadi, Constantinos Sioutas, Nicole Janssen, Anke H. Maitland - van der Zee, Ian Mudway, Flemming Cassee

**Affiliations:** a MRC Toxicology Unit, University of Cambridge, Cambridge, United Kingdom; b Amsterdam UMC, University of Amsterdam, Department of Respiratory Medicine, Meibergdreef 9, Amsterdam, the Netherlands; c Randall Centre of Cell and Molecular Biophysics, King's College London, London, United Kingdom; d University of Southern California, Department of Civil and Environmental Engineering, Los Angeles, CA, USA; e National Institute for Public Health and the Environment (RIVM), Bilthoven, Netherlands; f Environmental Research Group, Faculty of Medicine, School of Publuc Health, Imperial College London, London, United Kingdom; g National Institute of Health Research, Health Protection Research Unit in Environmental and Health, Faculty of Medicine, School of Public Health, Imperial College London, London, United Kingdom; h Institute for Risk Assessment Studies, Utrecht University, Utrecht, Netherlands

**Keywords:** Airport, Ultrafine particles, Metabolome, Oxidative stress, Biomarkers, Human exposure

## Abstract

**Background:**

Inflammation, oxidative stress and reduced cardiopulmonary function following exposure to ultrafine particles (UFP) from airports has been reported but the biological pathways underlying these toxicological endpoints remain to be explored. Urinary metabolomics offers a robust method by which changes in cellular pathway activity can be characterised following environmental exposures.

**Objective:**

We assessed the impact of short-term exposures to UFP from different sources at a major airport on the human urinary metabolome.

**Methods:**

21 healthy, non-smoking volunteers (aged 19–27 years) were repeatedly (2–5 visits) exposed for 5h to ambient air at Amsterdam Airport Schiphol, while performing intermittent, moderate exercise. Pre- to-post exposure changes in urinary metabolite concentrations were assessed via ^1^H NMR spectroscopy and related to total and source-specific particle number concentrations (PNC) using linear mixed effects models.

**Results:**

Total PNC at the exposure site was on average, 53,500 particles/cm^3^ (range 10,500–173,200) and associated with significant reductions in urinary taurine (−0.262 AU, 95% CI: −0.507 to −0.020) and dimethylamine concentrations (−0.021 AU, 95% CI: −0.040 to −0.067). Aviation UFP exposure accounted for these changes, with the reductions in taurine and dimethylamine associating with UFP produced during both aircraft landing and take-off. Significant reductions in pyroglutamate concentration were also associated with aviation UFP specifically, (−0.005 AU, 95% CI: −0.010 – <0.000) again, with contributions from both landing and take-off UFP exposure. While non-aviation UFPs induced small changes to the urinary metabolome, their effects did not significantly impact the overall response to airport UFP exposure.

**Discussion:**

Following short-term exposures at a major airport, aviation-related UFP caused the greatest changes to the urinary metabolome. These were consistent with a heightened antioxidant response and altered nitric oxide synthesis. Although some of these responses could be adaptive, they appeared after short-term exposures in healthy adults. Further study is required to determine whether long-term exposures induce injurious effects.

## Abbreviations

^1^H NMR: Proton nuclear magnetic resonance spectroscopy
ADMA: Asymmetric dimethylarginine
AU: Arbitrary unit
BC: Black carbon
CI: Confidence interval
CO: Carbon monoxide
CPC: Condensation particle counter
DDAH: Dimethylarginine dimethylaminohydrolase
DEP: Diesel exhaust particle
DMNA: Dimethylnitrosamine
FeNO: Fractional exhaled nitric oxide
FEV_1_: Forced expiratory volume
FVC: Forced vital capacity
HOCl: Hypochlorous acid
HSQC: Heteronuclear single quantum coherence
LAX: Los angeles international airport
NO: Nitric oxide
NOS: Nitric oxide synthases
NO_x_: Nitrogen oxides
Nrf2: Nuclear factor erythroid 2-related factor 2
PEF: Peak expiratory flow
PM: Particulate matter
PM2.5: PM2.5 fine particles (0.1–2.5 μm)
PM10: PM10 course particles 2.5–10 μm
PMF: Positive matrix factorization
PNC: Particle number concentrations
PPM: Parts per million
PURGE: Pre-saturation utilising relaxation gradients and echoes
REFs: Road traffic particulates
ROS: Reactive oxygen species
SEM: The standard error of the mean
SMPS: Scanning mobility particle sizer
SO_x_: Sulphur oxides
STOCSY: Statistical total correlation spectroscopy
TBEs: Tertiary-butyl ethers
TMAO: Trimethylamine-N-Oxide
TOCSY: Total correlation spectroscopy
TSP: Trimethylsilylpropanoic acid
UFP: Ultrafine particles
VOCs: Volatile organic compounds

## 1 Introduction

Global air transport has grown strongly over the past decades (Lee et al., 2009). In 2019, scheduled passenger numbers reached more than 4.5 billion and 61.3 million tonnes of cargo were transported by plane (International Air Transpo, 2020). Encouraged by our developing understanding of road emissions toxicity, concern has developed over the impacts that aviation and other airport emissions could have on human health. In addition to manoeuvring aircraft, auxiliary power units, ground service equipment and ground access vehicles are strong sources of nitrogen oxides (NO_x_), carbon monoxide (CO), volatile organic compounds (VOCs), sulphur oxides (SO_x_) and particulate matter (PM) at airports (Yang et al., 2018; Winther et al., 2015; Pirhadi et al., 2020; Simonetti et al., 2015).

Much of airport-originating PM falls within the ultrafine size range (PM < 0.1 μm) (Pirhadi et al., 2020) and is dominated by particles <20 nm in diameter based on the particle number concentrations (PNC) counts. This fraction is apportioned to primarily aircraft emissions (Pirhadi et al., 2020; Keuken et al., 2015). Concentrations of aviation-related PNCs have been detected at significantly elevated levels as far as 18 km downwind of airports (Keuken et al., 2015; Hudda et al., 2014, 2018; Rivas et al., 2020) affecting both total indoor and outdoor PNCs (Hudda et al., 2014, 2018). Resultantly the number of individuals exposed to aircraft -related emissions far exceeds airport personnel and passengers, extending to residents and workers in the urbanisations that neighbour airports.

Historically, studies of PM toxicity have focused primarily on the adverse impacts of exposure to PM10 and PM2.5 (Park et al., 2018; Gerlofs-Nijland et al., 2007, 2019; Kim et al., 2015; Khan and Strand, 2018). However, expansion of these studies to incorporate traffic-related UFPs has demonstrated the potential for UFPs to elicit greater toxicity (based on mass concentrations) and a higher likelihood to induce systemic effects compared with larger particles of the same composition. This is due to their small diameter, high surface area- to- mass ratio and high number concentration. These properties allow UFPs to adsorb greater quantities of redox-active metals and organic compounds and to deposit efficiently within the alveoli where they generate oxidative stress and inflammation, inhibit antimicrobial mechanisms and rapidly enter the surrounding tissue, avoiding clearance by airway macrophages (Kwon et al., 2020; Geiser et al., 2005; Lundborg et al., 2006; Li et al., 2003). Furthermore, small quantities of UFPs can cross the alveolar-capillary barrier, enter the bloodstream and translocate to secondary organs within hours of pulmonary exposure (Kreyling et al., 2002; Oberdörster et al., 2004; Brown et al., 2002).

Consistent with these observations from UFPs emitted by road-traffic, aviation-related UFPs have been shown to induce both airway and systemic inflammation *in vivo*. In mice, particles collected from a commercial airport and non-commercial airfield caused dose-dependent infiltration of the airways by neutrophils, lymphocytes and eosinophils during the first 24h of exposure (Bendtsen et al., 2019). Heightened concentrations of pro-inflammatory mediator IL-6 were also observed in the blood of asthmatic adults following a 2-h walk in the high UFP-zone surrounding Los Angeles International Airport (LAX) (Habre et al., 2018). Supported by evidence that exposure to UFP from Amsterdam Airport Schiphol associates with mild reductions in lung and cardiac function (decreased forced vital capacity and prolonged QTc intervals) in healthy young individuals (Lammers et al., 2020). While studies of adverse health effects and UFP exposure in airport workers remain scarce and inconclusive (Merzenich et al., 2021), the adverse effects that we see in these interventional and *in vitro* studies are consistent with those induced by diesel exhaust particles (DEP) (Bendtsen et al., 2021).Together with evidence that ultrafine DEP and aviation UFP have similar physicochemical properties (Bendtsen et al., 2021), these findings confirm the credibility of concern regarding aviation-related UFP exposure and health.

Expanding upon the observations from Amsterdam Airport Schiphol, this study employed untargeted metabolomics to identify response biomarkers that inform identification of potential causal adverse outcome pathways. Metabolomics captures the profile of small molecules that exist within a sample, and has been used to identify changes in cellular activity following exposure to fuel exhausts produced by road vehicles and ships (Oeder et al., 2015; Walker et al., 2019; Surowiec et al., 2016; Brower et al., 2016). Being global in design, these analyses identified components of adverse responses that were not captured previously by hypothesis-driven studies, including fuel- specific differences (Selley et al., 2019). For humans, urine is an especially favourable sample for metabolomic study of environmental exposures. As the primary route of excretion for cellular waste, urine is rich in metabolites and inclusive of pathway dysfunction markers from across the body (Bouatra et al., 2013). As a non-invasive and easily accessible sample, it also lends itself well to studies of large cohorts or repeat sampling.

Employing metabolomic urinalysis, this study explored the hypothesis that UFP from different airport-related sources (aviation, ground service vehicles and feeder highways) induces distinct changes to the urinary metabolome. Aiming to identify mechanistically informative markers of cellular responses to airport UFP exposure, we analysed the metabolic content of urine produced before and after short-term exposures to ambient air at Amsterdam Airport Schiphol, Netherlands.

## 2 Materials and methods

### 2.1 Study design and population

**Fig. 1:**
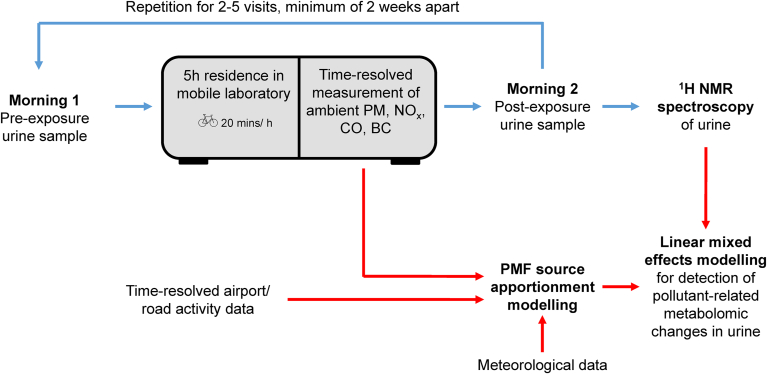
**Study design.** On 2 – 5 occasions, participants provided urine samples prior to spending 5h in a mobile laboratory at Amsterdam Airport Schiphol. A second urine sample was given at the 24h time point and the metabolomic content of each sample was characterised via ^1^H NMR. The output was combined with source apportioned pollutant measurements in a linear mixed effects model to identify changes in urinary metabolic content that relate to different air pollutant exposures at the airport. Exposure and experimental steps are denoted with blue arrows while data interrogation is represented by red arrows.

The design of this prospective, interventional study is detailed in Fig. 1. Healthy adults (n = 21) underwent 5h exposures to ambient air within a mobile laboratory at Amsterdam Airport Schiphol (Amsterdam, the Netherlands). Two to five repeat visits were made per participant, leaving a minimum of two weeks between visits to enable ablation of biological responses to exposure. Participants were university students, living in Amsterdam, > 2 km away from Schiphol airport and not within 300m away of a highway or road that was trafficked by > 10,000 vehicles per day. Participants were aged between 20 and 23 years, were predominantly female (81%) and had BMIs within healthy range (22.6 kg/m^2^ ± 2.4). All participants had normal cardiopulmonary function as determined by measurements of forced expiratory volume (FEV_1_), forced vital capacity (FVC), peak expiratory flow (PEF), fractional exhaled nitric oxide (FeNO), blood pressure, heart rate and oxygen saturation (presented previously in Table 2 of Lammers et al. 2020). Each participant provided first morning urine samples the day of exposure and again the next morning, an average of 18h after the end of exposure (minimum 12h, maximum 27h). Proton nuclear magnetic resonance spectroscopy (^1^H NMR) profiles were acquired for each urine sample to characterise changes in the urinary metabolome that related to UFP exposure at the airport.

### 2.2 Exposure

Detailed methods for participant exposures are provided by Lammers et al. (2020). Briefly, individuals remained for 5h within a mobile laboratory (14 m^3^), situated next to the airside of Amsterdam Airport Schiphol, ∼300m away from two runways, ∼500m from two highways, 10 km from the city and close to several large car parks. The laboratory was fitted with an airflow system to refresh the flow of ambient air in a uniform manner (∼400m (Yang et al., 2018)/h) for the duration of the exposure. Extensive air quality measurements were produced from the flow to characterise exposures for each individual at each visit. This varied due to differences in meteorological conditions (especially wind direction) and in runway use. By considering forecasted weather, we were able to schedule visits to include variation in UFP levels and source contributions (e.g. aviation and road traffic) for each individual. During the exposures, participants cycled at low intensity for 20 min/h and rested for the remainder of the time. Prior to exposures, participants were instructed not to consume alcohol or caffeine (for 24 and 12h respectively) as well as tobacco and non-pharmaceutical drugs for the duration of the study. Food and drinks were provided on the day of exposure to minimise intake of nitrate-rich foods but individual intake was not standardised or limited.

### 2.3 Exposure characterisation

Individual exposures to UFP emissions from aviation emissions (total, take-off only and landing only), non-aircraft airport vehicles (such as passenger buses, fuel tankers, baggage trucks and local airport traffic) and non-airport (urban background and road) sources were calculated as a 5h average for each exposure date using a Positive Matrix Factorization (PMF) source apportionment model. Details of the model and instruments used to collect the input data are provided by Pirhadi et al. (2020) (Pirhadi et al., 2020) and Lammers et al. (2020) (Lammers et al., 2020). To summarise the sources of the UFP, air within the exposure chamber was sampled continuously and subjected to measurement for particle number concentrations (PNCs) and PM mass (gravimetrically), carbon monoxide (CO), black carbon (BC) and nitrogen oxides (NO_x_). A water-based condensation particle counter (CPC) provided PNCs for total PM of ≤ ∼2.5 μm in diameter and a scanning mobility particle sizer (SMPS) was fitted to measure PNCs for size fractions between 6 and 225 nm in diameter. Particle masses were established using a tapered element oscillating microbalance while NO_x_ was measured with a chemiluminescence NO_x_ analyzer, CO with a gas filter correlation analyser and BC via optical absorption using an aethalometer. Meteorological conditions (temperature, wind speed and relative humidity) for the times of sampling were provided by the Royal Netherlands Meteorological Institute.

### 2.4 ^1^H NMR spectral acquisition

Samples were prepared by combining 540 μl urine with 60 μl phosphate buffer containing 0.1M trimethylsilylpropanoic acid (TSP) in 5 mm NMR tubes. Spectra were acquired with a 600 MHz AV-NEO spectrometer equipped with a triple resonance cryoprobe with ^1^H/^13^C/^15^N channels and a SampleJet for automation (all Bruker, UK). For each sample, a 1D ^1^H spectrum was acquired using the pre-saturation utilising relaxation gradients and echoes (PURGE) pulse sequence, optimised to reduce the effects of non-ideal gradients (Le Guennec et al., 2017). 64 scans were used, with an acquisition time of 2.62 s, a spectral width of 20.8 parts per million (ppm), 4 dummy scans and a relaxation delay of 4 s. Additionally, a Total Correlation Spectroscopy (TOCSY) spectrum and a heteronuclear single quantum coherence (HSQC) spectrum were acquired on one of the samples for identification purposes. For TOCSY, the Bruker pulse sequence “dipsi2gpphzs” was used, slightly modified to include presaturation, which 16 scans, 512 t1 increments, a spectral width of 13.7 ppm in both dimensions and a relaxation delay of 2 s. The HSQC spectrum was acquired using the Bruker pulse sequence “hsqcetgpsisp2.2”, with 32 scans, 512 t1 increments, a spectral width of 210 ppm in the 13C dimension and 20.8 ppm in the ^1^H dimension and a relaxation time of 2 s.

### 2.5 ^1^H NMR spectral processing and peak assignment

After acquisition, the 1D ^1^H spectra were processed with an exponential window function of 0.3 Hz before Fourier Transform, then phasing, calibration of the ppm scale to the TSP peak (0 ppm) and baseline correction with a polynomial function of order 2. Processed spectra were imported into Chenomx Profiler (Chenomx Inc, Canada) for annotation using a peak fitting technique. Statistical total correlation spectroscopy (STOCSY) analyses were performed using Matlab (Version R2019b, Mathworks, USA) to assist this process by identifying peaks that belonged to common parent molecules (r values > 0.8). Annotations were confirmed for feature metabolites by comparing peak signals within the HSQC spectrum with reference values published in the Human Metabolome Database (Human Metabolome Database, 2021). Integrals were calculated for individual peaks using Matlab, employing code that determined the size of the signal based on the area under the curve between peak minima and maxima. Peak integrals were normalised to those of creatinine signals from the same spectrum to account for variations in urinary concentration. All Matlab codes were developed within the Section of Computational and Systems Medicine, Imperial College, London.

### 2.6 Statistical analysis

Linear mixed effects models were used to (A) detect confounding variables in the dataset and (B) identify changes in urinary metabolite content that are related to pollutant exposure. These were performed in R Studio (version 1.1.463, USA) using the ‘lmer’ function of Package ‘lme4’. Throughout the analysis, relationships between metabolite signals and variables of interest (presented as regression coefficients) were considered statistically significant where the 95% confidence interval did not contain zero.

### 2.7 Confounding variables

To identify whether use of over-the counter pharmaceuticals (acetaminophen and ibuprofen, as detected within the spectra) induced changes to the urinary metabolome independent of pollutant exposure, pre-exposure data (pre) for each individual (i) and visit (j) was input into the following model:

Y_i,j_ = β_0_ ​+ ​Y_i,j,pre_ ​+ ​β_1_ E_j_ ​+ ​U_0i_ ​+ ​ε_i_

With Yi,j referring to the relationships between non-target variable and metabolomic change across the study, Ej represents a vector of the potentially confounding variables and Yi,j,pre, the metabolite signals produced from the pre-exposure spectra of each participant at each of their visits. β refers to population-average fixed effects; specifically, the average metabolite signal where all other co-variates are zero (β0) and the average signal relative to a 5-95th percentile (5-95p) increase in the variable of interest. The U0i is a random intercept produced from each individual's deviation from the study population's average metabolite signal with ε1 as the accompanying error term.

### 2.8 Pollutant-related metabolomic changes

Alterations were made to the confounding variable identification model to focus on changes in metabolite concentration that were caused by pollutant exposure and to correct for confounding co-exposures.

Y_i,j_ = β_0_ ​+ ​Y_i,j,pre-post_ ​+ ​β_1_E_j_ ​+ ​β_2_V_1,i,j_ ​+ ​β_3_V_2,j_ ​+ ​β_4_V_3,j_ ​+ ​U_0i_ ​+ ​ε_i_

Here, the model calculated the difference in metabolite signals for each individual at each visit (Y_i,j,pre-post_) whilst adjusting for vectors of pharmaceutical signals produced from the ^1^H NMR spectra (V_1,i,j_), environmental conditions (room temperature and humidity) for different visits (V_2,j_) and where appropriate, concentrations of secondary pollutants during different visits (V_3,j_), β_2_, β_3_ and β_4_ represent population- averages for these variables.

Pearson's correlation analyses were performed using GraphPad Prism 8 (GraphPad, California, USA) to explore the strength of relationships between metabolites that associated with exposure (referred to as feature metabolites). Correlations were considered ‘moderate’ or ‘strong’ where the Pearson's r value was ≥0.60 and 0.80, respectively (Akoglu, 2018), and the p value was ≤0.05.

## 3 Results

### 3.1 Individual particle exposures were predominantly contributed to by aviation emissions

In total, samples from 21 of the exposed participants were included for metabolomic profiling. Spectra from 1 participant were withdrawn from the analysis following peak annotation due to the presence of ethanol peaks in the urine. The remaining samples represented participation on 32 exposure days with each individual undergoing 2–5 exposures during the period between May and October 2018. Only 2 participants undertook two exposures (finishing the study early for personal reasons), with 13 participants undertaking four exposures and 6 participants undertaking five exposures due to extremely low exposures occurring on their first visit.

**Fig. 2:**
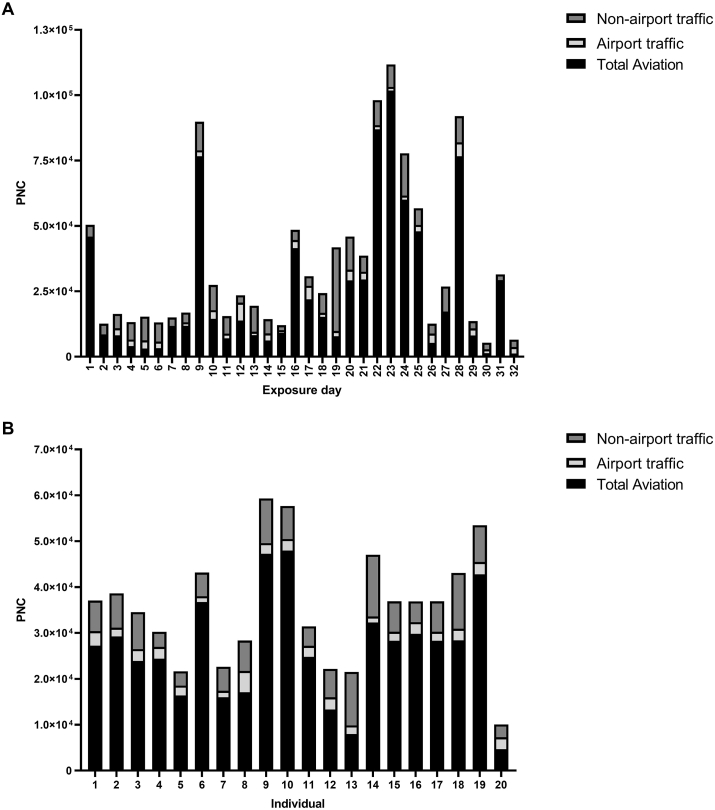
**PNC for total aviation, airport traffic and non-airport traffic sources during exposures**. Source apportioned 5h mean values are provided for each exposure event (A) as determined by PMF modelling (Pirhadi et al., 2020) and for each participant (presented as the mean of these values across their 2 – 5 exposures) (B).

As documented by Lammers et al. and Pirhadi et al.*,* 5 h averages of total PNC ranged between 10,500 and 173,200/cm^3^ at the exposure site (Lammers et al., 2020), with aviation activity contributing most to PNC exposure for the majority of the study period and individuals (Fig. 2A–B) (Pirhadi et al., 2020). Pearson's correlation analysis found no significant relationship between PNC from total aviation, airport traffic and non-airport traffic sources but as expected, total aviation PNC correlated strongly and positively with total PNC measurements, take – off PNC and landing PNC (r = 0.97, 0.97 and 0.89 respectively) (Table S1). Moderately strong positive correlations also existed between take-off and landing PNCs (r = 0.76) (Table S1).

### 3.2 ^1^H NMR spectra revealed substantial use of analgesics within the study population

**Fig. 3:**
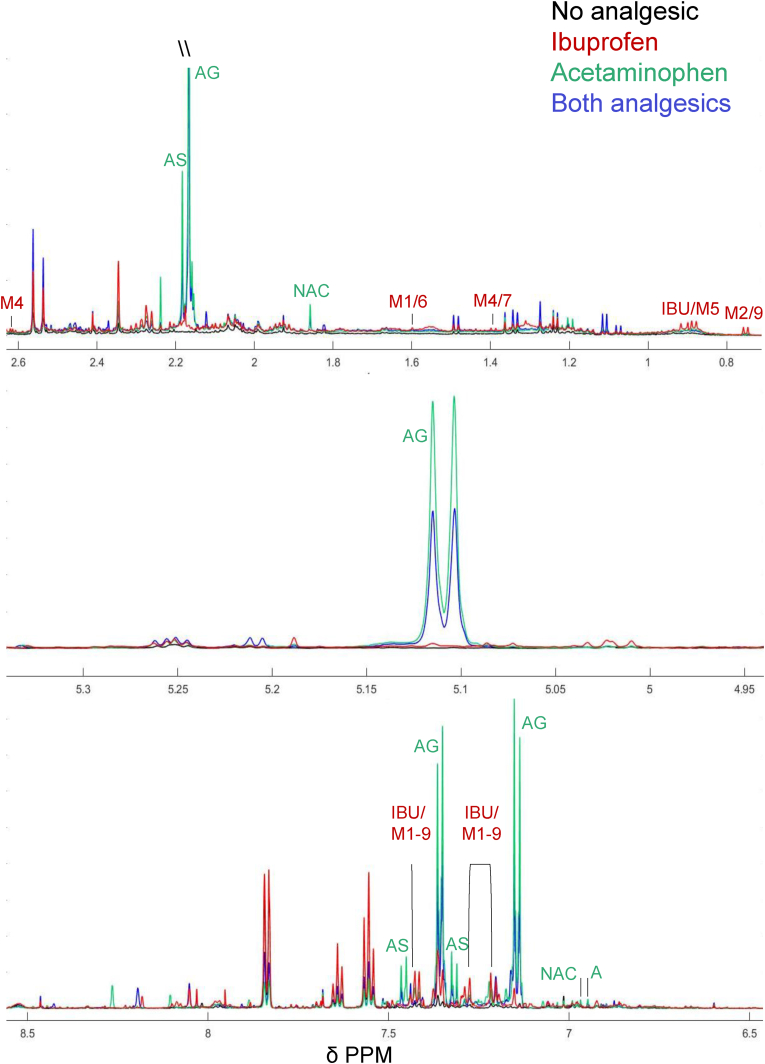
**Comparison of selected analgesic peak regions in the urinary**^**1**^**H NMR spectra of study participants prior to airport exposure.** As labelled, these regions display peaks relating to the presence of parent compounds or metabolites of acetaminophen or ibuprofen. Included spectra are representative of those that contain no analgesic peaks (black spectrum), ibuprofen related peaks (red spectrum), acetaminophen related peaks (green spectrum) or peaks relating to both analgesics (blue spectrum). Acetaminophen (A), acetaminophen sulfate (AS), acetaminophen glucuronide (AG), N-acetylcysteine (NAC), Ibuprofen (IBU), 1-hydroxy ibuprofen (Ibuprofen metabolite (M) 2), carboxy ibuprofen (M4), 2-hydroxy ibuprofen glucuronide (M6), carboxy ibuprofen glucuronide (M7), 1-hydroxy ibuprofen glucuronide (M9).

Peaks from 68 distinct, assignable metabolites were detected within the ^1^H NMR spectra alongside a further 54 peaks that could not be assigned using Chenomx profiling, HMBD searches or literature searches (Table S2). Of the assignable metabolites, 9 were produced during metabolism of commonly used, over-the-counter analgesics (ibuprofen and acetaminophen). As use of these analgesics were not exclusion criteria for the study, incidence of use by the study population was assessed using the visibility of the ibuprofen/ibuprofen-glucuronide peak at 0.74 ppm and the acetaminophen- glucuronide peaks at 5.10 ppm as markers of recent ibuprofen and acetaminophen consumption (Fig. 3). These peaks were selected because they did not exhibit overlap from other metabolites in the spectra. Visible ibuprofen/ibuprofen-glucuronide peaks, were present in 47 and 50% of pre- and post-exposure spectra (respectively), while visible acetaminophen glucuronide peaks were detected in 20 and 13% of pre- and post-exposure spectra. The presence of both analgesic peaks was detected in 12 and 5% of pre- and post-exposure spectra while only 32 and 41% of pre and post-exposure peaks contained no visible peaks for either metabolite.

Although pharmaceutical use is commonly observed in metabolomic analyses, the impact that therapeutic acetaminophen or ibuprofen use has on the endogenous urinary metabolome in humans has not been published. For the current study, linear modelling of the pre-exposure spectra demonstrated that urinary concentrations of trimethylamine-N-Oxide (TMAO), 3-aminoisobutyrate and glutamine were significantly elevated in association with acetaminophen use, and that citrate, glutamine, threonine, dimethylamine, alanine, TMAO, pyruvate, glutamate, lysine and N-acetylglutamate concentrations were significantly increased with ibuprofen uptake (Table 1). As such, urinary concentrations of acetaminophen and ibuprofen were input as confounding variables during modelling of emissions-related metabolomic change.

**Table 1:** Associations between Δ endogenous metabolites and xenobiotic metabolite concentrations.

Metabolite	Ibuprofen	Acetaminophen
	Coef. (95% CI)	Coef. (95% CI)
Citrate	**4.55 (2.30** – **6.90)**	0.17 (−1.63 – 2.03)
Glutamine	**1.69 (1.02 – 2.40)**	**0.866 (0.33 – 1.41)**
Threonine	**1.48 (1.09 – 1.88)**	0.34 (−0.04 – 0.72)
Dimethylamine	**1.48 (0.52 – 2.43)**	0.04 (−0.81 – 0.90)
Alanine	**1.42 (1.04 – 1.80)**	0.19 (−0.19 – 0.16)
TMAO	**1.39 (0.04 – 2.82)**	**1.19 (0.19** – **2.23)**
Unassigned at 2.33 ppm	**0.81 (0.36** – **1.44)**	0.32 (−0.04 – 0.67)
Pyroglutamate	**0.74 (0.45** – **1.05)**	0.33 (0.11 – 0.56)
Acetate/Phenylacetylglutamine	**0.54 (0.30** – **0.78)**	0.11 (−0.09 – 0.11)
3-Aminoisobutyrate	0.72 (−0.34 – 1.84)	**1.12 (0.32** – **1.95)**

Data are presented as coefficients (coef.) of the relationship between exposure and Δ in metabolite concentration (post-pre) with 95% confidence intervals (CI) (expressed as the range between lower and upper values). All coefficients are adjusted for room temperature and humidity. Numbers in bold represent significant relationships (p ≤ 0.05).

**Table 2:** Single pollutant models for associations between Δ urinary metabolites and major air pollutants at Schiphol Airport.

Metabolite	Total PNC (5-95p = 120,280 #/cm^3^)	PNC <20 nm (5-95p = 51,160 #/cm^3^)	PNC >50 nm (5-95p = 3,900 #/cm^3^)	Black carbon (5-95p = 1.4 μg/cm^3^)	NO_2_ (5-95p = 33.2 μg/cm^3^)	CO (5-95p = 250 μg/cm^3^)
	**Coef. (95% CI)**	**Coef. (95% CI)**	**Coef. (95% CI)**	**Coef. (95% CI)**	**Coef. (95% CI)**	**Coef. (95% CI)**
Taurine	**−0.263 (−0.507 – −0.020)**	**−0.298 (−0.550 – −4.709)**	−0.044 (−0.396 – 0.307)	−0.029 (−0.349 – 0.290)	−0.096 (−0.351 – 0.158)	−0.113 (−0.436 – 0.211)
Dimethylamine	**−0.023 (−0.040 – −0.067)**	**−0.023 (−0.040 – −0.067)**	0.006 (−0.018 – 0.029)	0.003 (−0.018 – 0.024)	−0.001 (−0.018 – 0.016)	−0.003 (−0.025 – 0.019)
Unassigned at 2.85 ppm	0.000 (−0.002 – 0.002)	−0.001 (−0.002 – 0.001)	0.000 (−0.002 – 0.003)	0.000 (−0.002 – 0.002)	0.001 (−0.003 – 0.002)	0.000 (−0.002 – 0.002)
3-Hydroxyisovalerate	−0.001 (−0.005 – 0.004)	0.000 (0.000 – 0.000)	−0.002 (−0.008 – 0.004)	−0.004 (−0.009 – 0.001)	**−0.005 (−0.009 – −0.001)**	−0.005 (−0.010 – 0.001)
3-Hydroxyisobutyrate	−0.002 (−0.005 – 0.001)	−0.002 (−0.005 – 0.002)	−0.002 (−0.006 – 0.002)	−0.001 (−0.005 – 0.003)	**−0.007 (−0.013 – −0.001)**	**−0.009 (−0.017 – −0.001)**
N-Acetylglutamine	0.000 (−0.015 – 0.015)	0.002 (−0.014 – 0.017)	0.001 (−0.022 – 0.023)	−0.007 (−0.029 – 0.013)	0.000 (−0.001 – 0.000)	**0.020 (0.002** – **0.038)**
Unassigned at 1.99 ppm	−0.001 (−0.004 – 0.002)	−0.001 (−0.004 – 0.003)	0.003 (−0.002 – 0.008)	0.003 (−0.001 – 0.000)	0.001 (−0.002 – 0.005)	**0.006 (0.001** – **0.010)**

Data are presented as coefficients (coef.) of the relationship between exposure and Δ in metabolite concentration (post-pre) with 95% confidence intervals (CI) (expressed as the range between lower and upper values). All coefficients are adjusted for urinary ibuprofen and paracetamol markers, room temperature and humidity. Total PNC refers to particles smaller than 2.5 μm in diameter, with a lower limit of 4 nm, as measured by a condensation particle counter and SMPS. Numbers in bold represent significant relationships (p ≤ 0.05).

### 3.3 Exposure to airport-derived particulates causes significant alterations to the endogenous urinary metabolome

Preliminary analysis determined that total PNC exposure was associated with significant reductions in urinary taurine and dimethylamine concentrations (−0.263 arbitrary units (AU, as a ratio with internal creatinine signal), 95% CI: −0.507 to −0.020 and −0.232 AU, 95% CI: −0.396 to −0.670, respectively). Size apportioned PNCs confirmed that these changes associated with exposure to PNC <20 nm but not PNC >50 nm. The strength of association between dimethylamine concentration and PNC <20 nm was of equal size to the relationship between dimethylamine concentration and total PNC, while a 0.035 AU increase in coefficient size was seen for the association between taurine concentration and PNC <20 nm exposure when compared with total PM exposure (Table 2). These observations indicate that PNC <20 nm, which associate with airplane emissions, were responsible for the changes. No other changes to the metabolome associated significantly with PNC <20 nm or PNC >50 nm or with carbon black exposure specifically (Table 2) but exposure to combustion-associated pollutant gases displayed small but significant associations with changes to the urinary metabolome. NO_2_ exposure related to small reductions in urinary 3-hydroxyisovalerate content (−0.005 AU, 95% CI: −0.009 to −0.001) as well as 3-hydroxyisobutyrate (−0.007 AU, 95% CI: −0.013 to −0.001). Exposure to CO also associated with reductions in 3-hydroxyisobutyrate concentration (−0.009 AU, 95% CI: −0.017 to −0.001) and increases in concentrations of N-acetylglutamine and an unassigned metabolite (0.020 AU, 95% CI: 0.002–0.038 and 0.006 AU, 95% CI: 0.001–0.010, respectively). Accounting for co-exposures to CO or NO_2_ had minimal impact on the strength of association between total PNC, PNC <20 nm and taurine or dimethylamine. No novel associations between exposure and metabolomic change were identified following the correction (Table S3).

### 3.4 Exposure to UFP from different airport-related sources induces distinct alterations to the endogenous urinary metabolome

The PMF model established that airport activities accounted for 79.3% of total PNC (46.1, 26.7 and 6.5% from aircraft departures, aircraft arrivals and ground service equipment (GSE)/local airport traffic respectively) while road traffic and urban background sources contributed 18% and 2.7% respectively (Pirhadi et al., 2020). Using this data, PNCs were assigned to three general emissions sources at Amsterdam Airport Schiphol; total aviation, airport traffic (from GSE and road traffic within the airport) and non-airport traffic (from the nearby highways and urban background). Consistent with the results above, the largest changes to the urinary metabolome of exposed participants were induced by total aviation PNC (5-95p = 73,485 particles/cm^3^). Here, exposure associated significantly with a 0.26 AU decrease in urinary taurine (95% confidence interval (CI): −0.503 to −0.023) as well as smaller but statistically significant decreases in dimethylamine (- 0.021 AU, 95% CI: −0.037 to −0.005) and pyroglutamate concentration (0.005 AU, 95% CI: −0.01- <0.00). Neither PNC produced by airport traffic or non-airport traffic associated with changes in these metabolite concentrations but PNC relating to airport traffic (5-95p = 5077 particles/cm^3^) did associate with significant yet small increases in urinary concentrations of methylguanidine (0.001 AU, 95% CI: >0.000–0.002) and decreases in 3-aminoisobutyrate (- 0.010 AU, 95% CI: −0.019 - - 0.001). In contrast, exposure to PNC produced by non-airport traffic (5-95p = 15290 particles/cm^3)^ associated with significant increases in urinary 3-aminoisobutyrate concentration (0.010 AU, 95% CI: 0.002–0.017) as well as small increases in carnosine/arginine (0.005 AU, 95% CI: 0.001–0.008) and ethanolamine/isethionate concentrations (0.005 AU, 95% CI: 0.001–0.010) and a reduction in isocitrate concentration (−0.003, 95% CI: −0.005 to −0.001) (Table 3). Adjustment of the single pollutant models to account for co-exposure to UFP from the remaining key sources (total aviation, airport traffic, non-airport traffic, as appropriate), did not cause noteworthy changes to the strength of associations with metabolite features (Table 4). This indicates that the changes to the metabolome that associate with each key feature were unlikely to have been contributed to by co-exposure to the others.

**Table 3:** Single pollutant models for associations between Δ urinary metabolites and UFP from airport-related sources**.**

Metabolite	Total aviation PNC (5-95p = 73485 #/cm^3^)	Airport traffic PNC (5-95p = 5077 #/cm^3^)	Non-airport traffic PNC (5-95p = 15290 #/cm^3^)
	**Coef. (95% CI)**	**Coef. (95% CI)**	**Coef. (95% CI)**
Taurine	**−0.263 (−0.503 – −0.023)**	0.035 (−0.271 – 0.342)	−0.029 (−0.269 – 0.211)
Dimethylamine	**−0.021 (−0.037 – −0.005)**	0.010 (−0.011 – 0.030)	−0.002 (−0.018 – 0.014)
Pyroglutamate	**−0.005 (−0.010 – < 0.000)**	−0.003 (−0.009 – 0.004)	0.003 (−0.002 – 0.008)
3-aminoisobutyrate	0.002 (−0.006 – 0.010)	**−0.010 (−0.019 – −0.001)**	**0.010 (0.002** – **0.017)**
Methylguanidine	−0.001 (−0.001 – < 0.000)	**0.001 (> 0.000** – **0.002)**	−0.001 (−0.001 – 0.000)
Isocitrate	0.001 (−0.001 – 0.003)	0.002 (<0.000 – 0.004)	**−0.003 (−0.005 – −0.001)**
Carnosine/arginine	0.002 (−0.002 – 0.006)	0.001 (−0.004 – 0.006)	**0.005 (0.001** – **0.008)**
Ethanolamine/isethionate	0.002 (−0.003 – 0.007)	0.002 (−0.004 – 0.008)	**0.005 (0.001** – **0.010)**

Data are presented as coefficients (coef.) of the relationship between exposure and Δ in metabolite concentration (post-pre) with 95% confidence intervals (CI). All coefficients are adjusted for urinary ibuprofen and paracetamol markers, room temperature and humidity. Numbers in bold represent significant relationships (p ≤ 0.05).

**Table 4:** Two pollutant models for associations between Δ urinary metabolites and UFP from key sources, accounting for co-exposures to UFP from the remaining key sources.

PNC source of interest:	Total aviation (5-95p = 73485 #/cm^3^)		Airport traffic (5-95p = 5077 #/cm^3^)		Non-airport traffic (5-95p = 15290 #/cm^3^)	
Co-exposing PNC source accounted for:	Airport traffic	Non-airport traffic	Total aviation	Non-airport traffic	Total aviation	Airport traffic
**Metabolite**	**Coef. (95% CI)**	**Coef. (95% CI)**	**Coef. (95% CI)**	**Coef. (95% CI)**	**Coef. (95% CI)**	**Coef. (95% CI)**
Taurine	**−0.262 (−0.502 – −0.022)**	**−0.270 (−0.516 – −0.024)**	0.021 (−0.276 – 0.319)	0.026 (−0.291 – 0.343)	0.023 (−0.195 – 0.241)	−0.027 (−0.253 – 0.198)
Dimethylamine	**−0.021 (−0.037 – −0.005)**	**−0.022 (−0.038 – −0.006)**	0.008 (−0.012 – 0.028)	0.008 (−0.012 – 0.030)	0.001 (−0.014 – 0.015)	−0.002 (−0.017 – 0.013)
Pyroglutamate	**−0.005 (−0.010 – < 0.000)**	**−0.006 (−0.011 – −0.001)**	−0.003 (−0.009 – 0.003)	−0.002 (−0.008 – 0.004)	0.004 (<0.000 – 0.009)	0.003 (−0.002 – 0.007)
3-Aminoisobutyrate	0.002 (−0.006 – 0.009)	0.000 (−0.008 – 0.004)	**−0.015 (−0.030 – −0.001)**	**−0.015 (−0.030 – <−0.000)**	**0.010 (0.004 – −0.017)**	**0.009 (0.003** – **0.016)**
Methylguanidine	−0.001 (−0.001 – >0.000)	0.003 (−0.008 – 0.014)	**0.001 (>0.000** – **0.002)**	0.001 (<0.000 – 0.002)	<0.000 (−0.001 – >0.000)	<0.000 (−0.012 – >0.000)
Isocitrate	0.001 (−0.001 – 0.003)	0.002 (0.000 – 0.004)	0.002 (<0.000 – 0.004)	0.001 (−0.001 – 0.003)	**−0.003 (−0.005 – −0.002)**	**−0.003 (−0.004 – −0.001)**
Carnosine/arginine	0.002 (−0.002 – 0.006)	0.001 (−0.003 – 0.005)	0.001 (−0.004 – 0.006)	0.002 (−0.003 – 0.007)	**0.004 (>0.000** – **0.008)**	**0.004 (>0.000** – **0.009)**
Ethanolamine/isethionate	0.002 (−0.003 – 0.007)	0.001 (−0.004 – 0.006)	0.002 (−0.004 – 0.008	0.004 (−0.003 – 0.010)	**0.004 (>0.000** – **0.008)**	**0.005 (>0.000** – **0.009)**

Data are presented as coefficients (coef.) of the relationship between exposure to key PNC sources and Δ in metabolite concentration (post-pre) following adjustment of the model for co-exposure to the remaining two key sources of PNC at the airport. Data are presented with 95% confidence intervals (CI). All coefficients are adjusted for urinary ibuprofen and paracetamol markers, room temperature and humidity. Numbers in bold represent significant relationships (p ≤ 0.05).

### 3.5 Landing and take-off- related UFP both contribute to changes in the urinary metabolome

Using the PMF source apportionment model, it was possible to explore relationships between urinary metabolomic changes and individual aircraft behaviours. Single pollutant models demonstrated that total PNCs for UFPs produced during take- off (5-95p = 56,130 particles/cm^3^) and landing (5-95p = 31,200 particles/cm^3^) could be associated with the significant changes in urinary dimethylamine concentration (−0.019 AU, 95% CI: −0.037 to −0.001 for take-off and −0.031 AU, 95% CI: −0.012 to −0.001 for landing). Compared to the relationship with total aviation PNC, reductions in urinary taurine concentrations were larger when associated with landing PNC specifically (−0.413 AU, 95% CI: −0.689 to −0.136). While not statistically significant, due to variation in response levels, reductions in urinary taurine concentration were also present overall, following association with take-off PNC (−0.224 AU, 95% CI -0.495 – 0.047). Similarly, reductions in urinary pyroglutamate associated significantly with take-off PNC (−0.006 AU, 95% CI: −0.012 to −0.001) but their association with landing PNC displayed too much variability to be considered statistically significant (−0.004 AU, 95% CI: −0.001 – 0.003) (Table 5). These associations remained robust following adjustment of the models for co-exposure to airport or non-airport traffic UFP (Table S4).

**Table 5:** Single pollutant models for associations between Δ urinary metabolites and PNC produced through take-off and landing.

Metabolite	Take-off PNC (5-95p = 56130 #/cm^3^)	Landing PNC (5-95p = 31200 #/cm^3^)
	**Coef. (95% CI)**	**Coef. (95% CI)**
Taurine	−0.224 (−0.495 – 0.047)	**−0.413 (-0.689 – −0.136)**
Dimethylamine	**−0.019 (-0.037 – −0.001)**	**−0.031 (-0.049 – −0.013)**
Pyroglutamate	**−0.006 (-0.012 – −0.001)**	−0.004 (−0.010 – 0.002)

Data are presented as coefficients (coef.) of the relationship between exposure and Δ in metabolite concentration (post-pre) with 95% confidence intervals (CI). All coefficients are adjusted for urinary ibuprofen and paracetamol markers, room temperature and humidity. Numbers in bold represent significant relationships (p ≤ 0.05).

### 3.6 Features of the metabolomic response to aviation UFP exposure may contribute to common biological processes

**Fig. 4:**
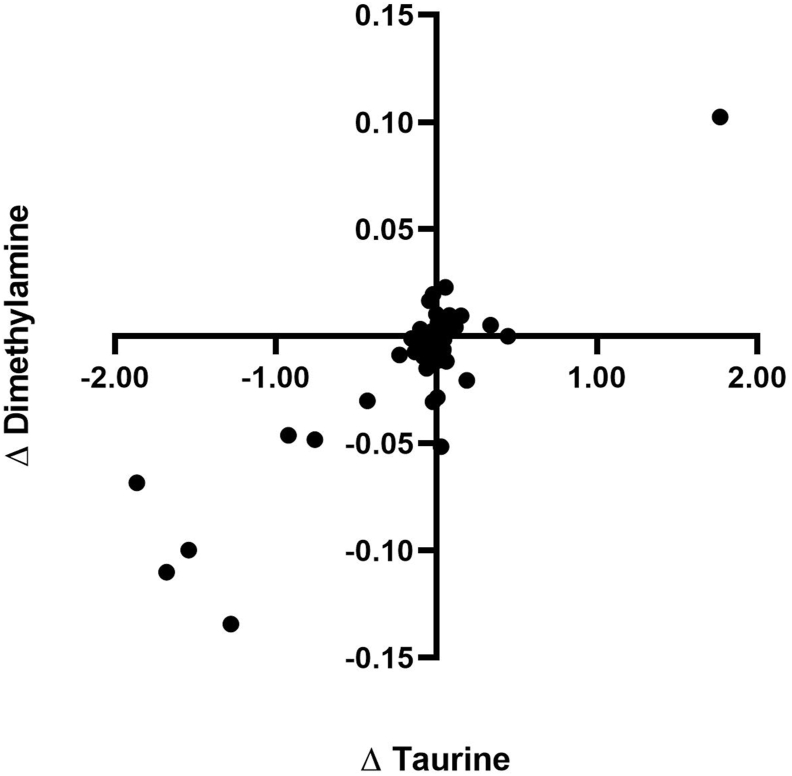
Pearson correlation between Δ urinary concentrations of taurine and Δ urinary concentrations of dimethylamine following total aviation UFP exposure. Analysis was performed using post- exposure minus pre- exposure values with each data point representing the outcome of one visit for individual participants.

In order to assign mechanistic meaning to metabolomic change, it is necessary to explore relationships between feature metabolites. With the three metabolites that associate with aviation UFP exposure, there was not sufficient input data to perform an appropriately powered pathway analysis. As such, Pearson's correlation analyses were performed to identify metabolites that could contribute to or be products of common biological processes. This method identified a strong, positive correlation (r = 0.88, p ≤ 0.001) between Δ dimethylamine and Δ taurine concentrations in post- and pre-exposure samples (Fig. 4). No strong nor significant correlations were found between Δ in taurine and Δ pyroglutamate concentration (r = 0.02) or between Δ dimethylamine and Δ pyroglutamate concentration (r = 0.02) (data not shown).

## 4 Discussion

Combining our cross-over intervention study of 21 healthy young adults with source apportionment modelling, we identified acute changes to the urinary metabolome that associate with exposure to UFP from distinct emission sources at Amsterdam Airport Schiphol. Metabolic signatures associating with aviation emissions dominated the response to total PNC and were characterised by significant reductions in urinary taurine, dimethylamine and pyroglutamate concentrations, consistent with increased utilisation or decreased synthesis of these metabolites.

Previously, exposure to airport UFPs has been associated with pulmonary and systemic inflammation (Bendtsen et al., 2019; Habre et al., 2018), oxidative stress (He et al., 2018) and reductions in cardiopulmonary function (Lammers et al., 2020). To our knowledge, this study is the first to assess responses to airport emissions at a global, biochemical level. Consistent with observations that airport UFPs have oxidative potential and induce reactive oxygen species (ROS) synthesis *in vitro* (He et al., 2018), several of the metabolites that associated with exposure to aviation UFPs in this study have been related to antioxidant responses to the imposition of oxidative stress.

The most pronounced of these changes was the reduction in urinary taurine which associated with UFP produced during aircraft landing and possibly take-off. The β-amino acid taurine, which is abundant in the cytosol of inflammatory and metabolically active cells, has been proposed to act as an indirect antioxidant via enhancement of classical antioxidant concentrations (Tabassum et al., 2006) and modulation of mitochondrial ROS generation (Ju et al., 1007). It also acts as an anti-inflammatory agent through its capacity to react with neutrophil-derived hypochlorous acid (HOCl) to form taurine chloramine (Kim and Cha, 2014). When taken up into cells at sites of inflammation, taurine chloramine promotes a broad spectrum xenobiotic and antioxidant response through activation of the Nuclear factor erythroid 2-related factor 2, (Nrf2) transcription factor (Kim and Cha, 2014).

Decreased taurine concentrations have been measured in the BALF of rats following ZnO inhalation, reflecting enhanced antioxidant activity within the pulmonary tissue. Supporting the suggestion that landing UFPs also triggered this protective response, taurine has been shown to alleviate oxidative stress, pro-inflammatory cytokine secretion, inflammatory cell recruitment, mitochondrial dysregulation, autophagy and emphysema in mouse lung following exposure to DEP or 1-nitropyrene (Kim et al.; Li et al., 1073). It is difficult to hypothesise why the observed change in taurine concentration associated more robustly with landing UFP. To date, no considerable differences have been reported in the composition of emissions produced during take-off and landing (Shirmohammadi et al., 2017). Although landing particles did account for the majority of UFP <20 nm at our sampling site (Pirhadi et al., 2020), their concentrations were strongly correlated with those of take-off particles (r = 0.76), creating the possibility that the observed differences in effect size and significance were artefacts of collinearity within the model. While individuals are unlikely to only be exposed to landing UFP at an airport, confirming this observation and understanding its cause, could have bearing on future aviation engineering.

**Fig. 5:**
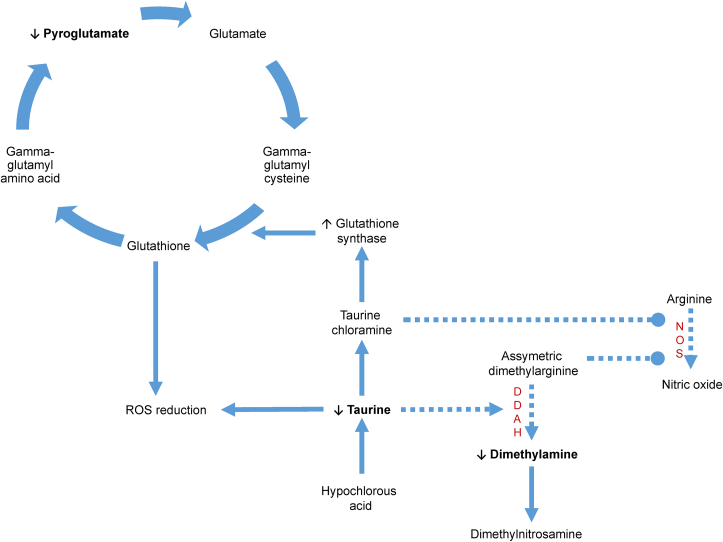
**Hypothesised interplay of altered pathway activity following exposure to aviation UFPs.** Reductions in urinary pyroglutamate reflect increased demand for glutathione synthesis which is contributed to by conversion of taurine to taurine chloramine with downstream, Nrf2-mediated induction of glutathione synthase expression. Taurine availability also diminishes due to the role of taurine as an inhibitor of ROS generation, leading to decreased DDAH agonism and dimethylamine synthesis. Resultant accumulations of ADMA, combined with increased availability of taurine chloramine inhibit NOS activity, resulting in reduced nitric oxide synthesis. Increased conversion of dimethylamine to nitrosdimethylamine may also contribute to reductions in dimethylamine concentration. Dotted and solid arrows represent reduction and enhancement of reactions (respectively). Dimethylarginine dimethylaminohydrolase (DDAH), nitric oxide synthase (NOS).

Exposure to both landing and take-off related UFPs induced reductions in urinary pyroglutamate. Pyroglutamate is produced in the γ-glutamyl cycle as a precursor to glutathione (GSH) (Lord and Bralley, 2008). A decrease in urinary pyroglutamate is therefore consistent with increased cellular GSH synthesis as an adaptive response to the imposition of oxidative stress. This aligns with a potential role for taurine chloramine in promoting GSH synthesis through Nrf2-mediated up-regulation of glutathione synthase expression (Steele et al., 2013) (Illustrated in Fig. 5). This hypothesis does require experimental confirmation, but in the context of the previous literature demonstrating the capacity for UFP to initially deplete antioxidants (Walker et al., 2019) and subsequently induce protective, adaptive responses (Li et al., 2004), the observed relationships do illustrate the utility of metabolomics in generating novel, testable hypotheses to explore causal links between pollutants and adverse responses. It is important to note however, that our results reflect only responses to short term exposures in healthy individuals and there remains a need to understand the impact of recurrent or longer exposures in relation to chronic disease development and exacerbation (Downward et al., 2018).

As well as pyroglutamate, UFPs produced during take-off and landing were also associated with reductions in urinary dimethylamine. In humans, dimethylamine is produced endogenously by the enzyme dimethylarginine dimethylaminohydrolase (DDAH) during hydrolysis of asymmetric dimethylarginine (ADMA) (Tsikas, 2020) and through microbial catabolism of dietary choline (Zeisel et al., 1985). In health, dimethylamine is excreted via the urine in the upper μM range (Tsikas, 2020), with a small fraction converted to dimethylnitrosamine (DMNA). Reduced urinary dimethylamine concentrations may therefore reflect enhanced DMNA synthesis or reduced DDAH activity. As DMNA exerts genotoxicity in mammalian cell lines and rodents and is hypothesised to act similarly in humans (International Agency for, 1978; Liteplo and Meek, 2001), this warrants further investigation.

As ADMA is an inhibitor of nitric oxide (NO) synthases (NOS), reduced conversion to dimethylamine could also result in decreased NO synthesis. Supporting this hypothesis, increased ADMA and NO precursors have been measured in the plasma of individuals exposed to highway UFPs (Walker et al., 2019) and reduced NO synthesis has been observed in human aortic endothelial cells following exposure to urban UFPs (Du et al., 2013). As well as impacting vascular tone, airway responsiveness and inflammatory cell function, reduced NO availability results in QT interval prolongation and reduced lung function (23,24). While cardiopulmonary function was not measured at the same time as urine sampling for this study, reductions in FVC and QT interval prolongation did associate with aviation UFP exposure in our cohort 4h post-exposure (Lammers et al., 2020). Strong positive associations (r = 0.88) were observed between the reductions in dimethylamine concentrations and reductions in taurine concentrations, suggesting some degree of interaction between the implicated metabolic pathways (Fig. 5). As DDAH is agonised by taurine (Pasaoglu et al., 2014; Tan et al., 2007) one explanation could be that the particle-induced decrease in taurine availability led to reduced DDAH activity via less agonism (Fig. 5). Like ADMA, taurine chloramine inhibits NOS activity during inflammation (Kim and Cha, 2014; Barua et al., 2001), supporting the plausibility of interactions between the two pathways following UFP exposure (Fig. 5).

Ibuprofen and acetaminophen metabolites were present in approximately 50% of spectra, making correction for their use, a necessity for our model. Like airport UFPs, these metabolites associated with changed urinary concentrations of dimethylamine, pyroglutamate, 3-aminoisobutyrate and mitochondrial metabolism markers, indicating their potential to mask our responses of interest. While many studies prohibit use of analgesics, this was not feasible for our six-month study period. As a result of correcting for their use post-exposure, we add to our outcomes, a preliminary characterisation of how therapeutic doses of ibuprofen and acetaminophen impact the human urinary metabolome. Until now, study of the impacts that these pharmaceuticals have on the human metabolome has been limited to the contexts of overdose and hepatotoxicity (30–34))

It must be noted that the participants of this study were predominantly female (81%) and were all young individuals with ‘healthy’ BMI and cardiopulmonary function who live in areas without high levels of traffic pollution. As such, we cannot presume that the hypothesised mechanisms of aviation UFP toxicity reflect the responses of individuals who do not fit these criteria. As examples, metabolomic responses to traffic-related PM exposure are shown to be influenced by asthmatic status (arginine-related pathways) (Liang et al., 2019) and by sex and obesity (non-esterified fatty acid metabolism) (Chen et al., 2019). It is therefore important that the hypothesised impacts of aviation UFP on the urinary metabolome are validated in larger, more diverse cohorts, especially those that are inclusive of established vulnerable groups.

## 5 Conclusions

In this study, we have for the first time, demonstrated a clear distinction between the urinary metabolomic signatures that accompany exposure to aviation UFPs and those that associate with other UFP sources at a major airport. From the metabolic features identified, the direction of their relationship with the exposure estimates and pre-existing knowledge base on UFP toxicity, we have elaborated a series of potential testable hypotheses based on the (A) increased utilisation of taurine and induction of an adaptive antioxidant response, including increased synthesis of GSH and (B) modulation of nitric oxide production via enhanced dimethylarginine dimethylaminohydrolase activity.

There remain outstanding questions as to whether the hypothesised responses are informative to an understanding of longer-term or repeat exposures, especially within established vulnerable groups. Considering however, that these responses are consistent with effects induced by road-side levels of traffic particulates (Oeder et al., 2015; Walker et al., 2019; Törnqvist et al., 2007), which have established links with adverse health (Khan and Strand, 2018; Sinharay et al., 2018; Adar et al., 2007; Zhou et al., 2015; Kan et al., 2007), and that airport particulates have been found to induce similar acute phase, inflammatory and genotoxic responses to DEP in mice (Bendtsen et al., 2019), we believe that the hypotheses merit further exploration. Additionally, our findings, just as previous studies of UFP exposure, emphasise the importance of UFP monitoring networks for a comprehensive examination of long-term UFP exposures and adverse health outcomes, especially in near-source environments such as major airports, to determine threshold levels and support UFP regulations (Baldauf et al., 2016).

## Ethics

The study protocol was reviewed and approved by the Medical Ethical Committee (METC) of the Amsterdam Medical Centre (Amsterdam, the Netherlands) and was registered at the Dutch Trial Register (identifier NTR 6955, www.trialregister.nl/). All participants provided informed consent.

## Funding

The field work was funded by the Netherlands Ministry of Infrastructures and Water Management as part of grant M240045 and commissioned to the National Institute for Public Health and the Environment. In addition, funding for data acquisition and analysis was provided by the 10.13039/501100000265Medical Research Council through a 10.13039/100014013UKRI Innovation/Rutherford Fund Fellowship grant (RG95376), awarded to LS and by the 10.13039/100010269Wellcome Trust and 10.13039/501100000274British Heart Foundation (ref. 202767/Z/16/Z and IG/16/2/32273, awarded to the King's Centre for Biomolecular Spectroscopy). IM is part funded by the 10.13039/501100000272National Institute for Health Research (NIHR) Health Protection Research Unit in Environmental Exposures and Health, a partnership between 10.13039/501100002141Public Health England and 10.13039/501100000761Imperial College London. The views expressed are those of the author(s) and not necessarily those of the NIHR, Public Health England or the Department of Health and Social Care.

## Declaration of competing interest

None.

## References

[bib1] Adar S.D. (2007). Focused exposures to airborne traffic particles and heart rate variability in the elderly. Epidemiology.

[bib2] Akoglu H. (2018). User's guide to correlation coefficients. Turkish.J. Emerg. Med..

[bib4] Baldauf R.W. (2016).

[bib5] Barua M., Liu Y., Quinn M.R. (2001). Taurine chloramine inhibits inducible nitric oxide synthase and TNF-α Gene expression in activated alveolar macrophages: decreased NF-κB activation and IκB Kinase activity. J. Immunol..

[bib6] Bendtsen K.M. (2019). Airport emission particles: exposure characterization and toxicity following intratracheal instillation in mice. Part. Fibre Toxicol..

[bib7] Bendtsen K.M., Bengtsen E., Saber A.T., Vogel U. (2021). A review of health effects associated with exposure to jet engine emissions in and around airports. Environ. Health: A Global Access Science Source.

[bib8] Bouatra S. (2013). The human urine metabolome. PLoS One.

[bib9] Brower J.B. (2016). Metabolomic changes in murine serum following inhalation exposure to gasoline and diesel engine emissions. Inhal. Toxicol..

[bib10] Brown J.S., Zeman K.L., Bennett W.D. (2002). Ultrafine particle deposition and clearance in the healthy and obstructed lung. Am. J. Respir. Crit. Care Med..

[bib12] Chen Z. (2019). Near-roadway air pollution exposure and altered fatty acid oxidation among adolescents and young adults – the interplay with obesity. Environ. Int..

[bib13] Downward G.S. (2018). Long-term exposure to ultrafine particles and incidence of cardiovascular and cerebrovascular disease in a prospective study of a Dutch cohort. Environ. Health Perspect..

[bib14] Du Y. (2013). Ambient ultrafine particles reduce endothelial nitric oxide production via S-glutathionylation of eNOS. Biochem. Biophys. Res. Commun..

[bib15] Geiser M. (2005). Ultrafine particles cross cellular membranes by nonphagocytic mechanisms in lungs and in cultured cells. Environ. Health Perspect..

[bib16] Gerlofs-Nijland M.E. (2007). Toxicity of coarse and fine particulate matter from sites with contrasting traffic profiles. Inhal. Toxicol..

[bib17] Gerlofs-Nijland M.E. (2019). Inhalation toxicity profiles of particulate matter: a comparison between brake wear with other sources of emission. Inhal. Toxicol..

[bib18] Habre R. (2018). Short-term effects of airport-associated ultrafine particle exposure on lung function and inflammation in adults with asthma. Environ. Int..

[bib19] He R.W., Shirmohammadi F., Gerlofs-Nijland M.E., Sioutas C., Cassee F.R. (2018). Pro-inflammatory responses to PM 0.25 from airport and urban traffic emissions. Sci. Total Environ..

[bib20] Hudda N., Gould T., Hartin K., Larson T.V., Fruin S.A. (2014). Emissions from an international airport increase particle number concentrations 4-fold at 10 km downwind. Environ. Sci. Technol..

[bib21] Hudda N., Simon M.C., Zamore W., Durant J.L. (2018). Aviation-related impacts on ultrafine particle number concentrations outside and inside residences near an airport. Environ. Sci. Technol..

[bib22] Human metabolome Database. https://hmdb.ca/.

[bib23] International Agency for Research on Cancer (1978). IARC publications Website - radiation. *Some N-Nitroso Compounds*: IARC Monogr. Eval. Carcinog. Risk Chem. Humans.

[bib24] International Air Transport Association (2020).

[bib26] Ju, C., Junichi, J. A. & Schaffer, S. Mechanism underlying the antioxidant activity of taurine: prevention of mitochondrial oxidant production. 10.1007/s00726-011-0962-7. 21691752

[bib27] Kan H. (2007). Traffic exposure and lung function in adults: the Atherosclerosis Risk in Communities study. Thorax.

[bib28] Keuken M.P., Moerman M., Zandveld P., Henzing J.S., Hoek G. (2015). Total and size-resolved particle number and black carbon concentrations in urban areas near Schiphol airport (The Netherlands). Atmos. Environ..

[bib29] Khan R.K., Strand M.A. (2018). Road dust and its effect on human health: a literature review. Epidemiology and health.

[bib30] Kim C., Cha Y.N. (2014). Taurine chloramine produced from taurine under inflammation provides anti-inflammatory and cytoprotective effects. Amino Acids.

[bib31] Kim, J. et al. Taurine treatment reduces oxidative stress and pulmonary inflammation by taurine a murine asthma model asthma induced by house dust and diesel particulate matter. Faseb. J. 25, 613.5-613.5.

[bib32] Kim K.H., Kabir E., Kabir S. (2015). A review on the human health impact of airborne particulate matter. Environ. Int..

[bib33] Kreyling W.G. (2002). Translocation OF ultrafine insoluble iridium particles from lung epithelium to extrapulmonary organs IS size dependent but very low. J. Toxicol. Environ. Health Part A.

[bib34] Kwon H.S., Ryu M.H., Carlsten C. (2020). Ultrafine particles: unique physicochemical properties relevant to health and disease. Exp. Mol. Med..

[bib35] Lammers A. (2020). Effects of short-term exposures to ultrafine particles near an airport in healthy subjects. Environ. Int..

[bib36] Le Guennec A., Tayyari F., Edison A.S. (2017). Alternatives to nuclear overhauser enhancement spectroscopy presat and carr-purcell-Meiboom-Gill presat for NMR-based metabolomics. Anal. Chem..

[bib37] Lee David, Fahey David, Forster Piers, Newton Peter, Wit Ron, Lim Liam, Owen Bethan, Sausen R. (2009). Aviation and global climate change in the 21st century | Elsevier Enhanced Reader. Atmos. Environ..

[bib38] Li, X. et al. Taurine ameliorates particulate matter-induced emphysema by switching on mitochondrial NADH dehydrogenase genes. 10.1073/pnas.1712465114. PMC5692577 29078374

[bib39] Li N. (2003). Ultrafine particulate pollutants induce oxidative stress and mitochondrial damage. Environ. Health Perspect..

[bib40] Li N. (2004). Nrf2 is a key transcription factor that regulates antioxidant defense in macrophages and epithelial cells: protecting against the proinflammatory and oxidizing effects of diesel exhaust chemicals. J. Immunol..

[bib41] Liang D. (2019). Perturbations of the arginine metabolome following exposures to traffic-related air pollution in a panel of commuters with and without asthma. Environ. Int..

[bib42] Liteplo R.G., Meek M.E. (2001). *N* -nitrosodimethylamine: hazard characterization and exposure–response analysis. J. Environ. Sci. Heal. Part C.

[bib43] Lord R.S., Bralley J.A. (2008). Clinical applications of urinary organic acids. Part 1: detoxification markers. Alternative Med. Rev..

[bib44] Lundborg M. (2006). Aggregates of ultrafine particles impair phagocytosis of microorganisms by human alveolar macrophages. Environ. Res..

[bib45] Merzenich H. (2021). Air pollution and airport apron workers: a neglected occupational setting in epidemiological research. Int. J. Hyg Environ. Health.

[bib46] Oberdörster G. (2004). Translocation of inhaled ultrafine particles to the brain. Inhal. Toxicol..

[bib47] Oeder S. (2015). Particulate matter from both heavy fuel oil and diesel fuel shipping emissions show strong biological effects on human lung cells at realistic and comparable in vitro exposure conditions. PLoS One.

[bib48] Park M. (2018). Differential toxicities of fine particulate matters from various sources. Sci. Rep..

[bib49] Pasaoglu O.T. (2014). The effect of taurine on the relationship between NO, ADMA and homocysteine in Endotoxin-mediated inflammation in HUVEC cultures. Inflammation.

[bib50] Pirhadi M. (2020). Relative contributions of a major international airport activities and other urban sources to the particle number concentrations (PNCs) at a nearby monitoring site. Environ. Pollut..

[bib51] Rivas I. (2020). Source apportionment of particle number size distribution in urban background and traffic stations in four European cities. Environ. Int..

[bib52] Selley L., Phillips D.H., Mudway I. (2019). The potential of omics approaches to elucidate mechanisms of biodiesel-induced pulmonary toxicity. Part. Fibre Toxicol..

[bib53] Shirmohammadi F. (2017). Emission rates of particle number, mass and black carbon by the Los Angeles International Airport (LAX) and its impact on air quality in Los Angeles. Atmos. Environ..

[bib54] Simonetti I., Maltagliati S., Manfrida G. (2015). Air quality impact of a middle size airport within an urban context through EDMS simulation. Transport. Res. Transport Environ..

[bib55] Sinharay R. (2018). Respiratory and cardiovascular responses to walking down a traffic-polluted road compared with walking in a traffic-free area in participants aged 60 years and older with chronic lung or heart disease and age-matched healthy controls: a randomised, crossover study. Lancet.

[bib56] Steele M.L. (2013). Effect of Nrf2 activators on release of glutathione, cysteinylglycine and homocysteine by human U373 astroglial cells. Redox Biol.

[bib57] Surowiec I. (2016). Multi-platform metabolomics assays for human lung lavage fluids in an air pollution exposure study. Anal. Bioanal. Chem..

[bib58] Tabassum H., Rehman H., Banerjee B.D., Raisuddin S., Parvez S. (2006). Attenuation of tamoxifen-induced hepatotoxicity by taurine in mice. Clin. Chim. Acta.

[bib59] Tan B. (2007). Taurine protects against low-density lipoprotein-induced endothelial dysfunction by the DDAH/ADMA pathway. Vasc. Pharmacol..

[bib60] Törnqvist H. (2007). Persistent endothelial dysfunction in humans after diesel exhaust inhalation. Am. J. Respir. Crit. Care Med..

[bib61] Tsikas D. (2020). Urinary dimethylamine (DMA) and its precursor asymmetric dimethylarginine (ADMA) in clinical Medicine, in the context of nitric oxide (NO) and beyond. J. Clin. Med..

[bib62] Walker D.I. (2019). Metabolomic assessment of exposure to near-highway ultrafine particles. J. Expo. Sci. Environ. Epidemiol..

[bib63] Winther M. (2015). Emissions of NOx, particle mass and particle numbers from aircraft main engines, APU's and handling equipment at Copenhagen Airport. Atmos. Environ..

[bib64] Yang X., Cheng S., Lang J., Xu R., Lv Z. (2018). Characterization of aircraft emissions and air quality impacts of an international airport. J. Environ. Sci. (China).

[bib65] Zeisel S.H., DaCosta K.A., Fox J.G. (1985). Endogenous formation of dimethylamine. Biochem. J..

[bib66] Zhou F. (2015).

