# Supplementary material for: Alterations to the urinary metabolome following semi-controlled short exposures to ultrafine particles at a major airport
Source: Int J Hyg Environ Health. 2021 Aug;237:113803. doi: 10.1016/j.ijheh.2021.113803 (PMC8504201; doi:10.1016/j.ijheh.2021.113803)
Supplement: Multimedia component 1 [file mmc1.docx]

**Alterations to the urinary metabolome following semi-controlled short exposures to ultrafine particles at a major airport**

**Liza Selley**, Ariana Lammers, Adrien Le Guennec, Constantinos Sioutas, Nicole Janssen, Anke-Hilse Maitland - van der Zee, Ian Mudway, Flemming Cassee

**Supplementary material**

| **Table S1:** Correlation matrix for all pollutants, particle size ranges and room conditions measured during 5 h exposures | | | | | | | | | | | | | | |
| --- | --- | --- | --- | --- | --- | --- | --- | --- | --- | --- | --- | --- | --- | --- |
|  | **PNC^a^** | **PM** | **BC** | **NO_2_** | **CO** | **Total aviation** | Take-off | Landing | **Total traffic** | Airport traffic | Non-airport traffic | **Temp** | **RH** |  |
| **PNC^a^** |  |  |  |  |  |  |  |  |  |  |  |  |  |  |
| **PM** | -0.12 |  |  |  |  |  |  |  |  |  |  |  |  |  |
| **BC** | 0.18 | 0.45 |  |  |  |  |  |  |  |  |  |  |  |  |
| **NO_2_** | 0.41 | 0.17 | **0.77** |  |  |  |  |  |  |  |  |  |  |  |
| **CO** | 0.08 | 0.34 | 0.60 | 0.58 |  |  |  |  |  |  |  |  |  |  |
| **Total aviation** | **0.97** | -0.15 | 0.15 | 0.37 | 0.06 |  |  |  |  |  |  |  |  |  |
| Take-off | **0.95** | -0.09 | 0.20 | 0.41 | 0.05 | **0.97** |  |  |  |  |  |  |  |  |
| Landing | **0.85** | -0.24 | 0.02 | 0.23 | 0.08 | **0.89** | **0.76** |  |  |  |  |  |  |  |
| **Total traffic** | 0.34 | 0.40 | 0.30 | 0.19 | 0.05 | 0.25 | 0.33 | 0.04 |  |  |  |  |  |  |
| Airport traffic | -0.01 | 0.09 | 0.26 | 0.38 | 0.26 | 0.03 | 0.10 | -0.11 | 0.22 |  |  |  |  |  |
| Non-airport traffic | 0.35 | 0.39 | 0.24 | 0.09 | -0.02 | 0.24 | 0.32 | 0.07 | 0.96 | -0.04 |  |  |  |  |
| **Temp** | -0.03 | 0.27 | 0.08 | 0.05 | -0.05 | 0.04 | 0.17 | -0.20 | 0.42 | 0.42 | 0.31 |  |  |  |
| **RH** | 0.04 | 0.36 | 0.41 | 0.29 | 0.55 | 0.03 | 0.08 | -0.08 | 0.09 | 0.13 | 0.05 | 0.29 |  |  |
| Pearson correlations with in bold R >0.70; PNC = particle number concentration detected by a condensation particle counter (CPC) with d_50_ = 4 nm; PM = particulate matter; BC = black carbon; NO_2_ = nitric oxide; CO = carbon monoxide; Temp = temperature; RH = relative humidity; Total aviation (also subdivided into “take-off” and “landing”) and total traffic (also subdivided into “airport traffic” and “road taffic”) are different sources of PNC. This table has been partly published previously (Lammers *et al.,* [doi.org/10.1016/j.envint.2020.105779](https://doi.org/10.1016/j.envint.2020.105779)). | | | | | | | | | | | | | | |

**Table S2: Locations of metabolite peaks within ^1^H NMR spectra**

| **Peak minimum (ppm)** | **Peak maximum (ppm)** | **Metabolite** |
| --- | --- | --- |
| 0.735 | 0.765 | 1-hydroxy ibuprofen glucuronide |
| 0.83 | 0.84 | 2-hydroxyisovalerate |
| 0.875 | 0.893 | Ibuprofen/ ibuprofen glucuronide |
| 1.035 | 1.06 | Valine |
| 1.06 | 1.09 | Carboxy ibuprofen/ carboxy ibuprofen glucuronide |
| 1.09 | 1.12 | 3-hydroxyisobutyrate |
| 1.12 | 1.13 | 4-deoxyerythreonic acid |
| 1.134 | 1.15 | Acetaminophen |
| 1.15 | 1.17 | U1 |
| 1.27 | 1.28 | 3-hydroxyisovalerate |
| 1.32 | 1.35 | Threonine |
| 1.36 | 1.38 | 2-hydroxyisobutyrate |
| 1.43 | 1.44 | U2 |
| 1.44 | 1.45 | Acetoin |
| 1.46 | 1.51 | Alanine |
| 1.89 | 1.92 | Acetate and phenylacetylglutamine |
| 1.92 | 1.93 | Acetate |
| 1.95 | 1.97 | Isoeugenol |
| 1.98 | 2 | U3 |
| 2.02 | 2.03 | U4 |
| 2.03 | 2.05 | Pyroglutamate |
| 2.33 | 2.36 | U5 |
| 2.43 | 2.51 | Glutamine |
| 2.52 | 2.57 | Citrate |
| 2.6 | 2.64 | 3-aminoisobutyrate |
| 2.71 | 2.74 | Dimethylamine |
| 2.78 | 2.79 | 2-hydroxyibuprofen |
| 2.795 | 2.805 | 2-hydroxyibuprofen glucuronide |
| 2.805 | 2.815 | U6 |
| 2.82 | 2.85 | Methylguanidine |
| 2.85 | 2.86 | U7 |
| 2.86 | 2.87 | U8 |
| 2.88 | 2.9 | U9 |
| 2.9 | 2.91 | Trimethylamine |
| 2.91 | 2.92 | U10 |
| 2.925 | 2.94 | N,N-dimethylglycine |
| 2.97 | 2.99 | Isocitric acid |
| 2.995 | 3.01 | U11 |
| 3.01 | 3.03 | U12 |
| 3.044 | 3.08 | Creatine |
| 3.115 | 3.125 | U13 |
| 3.125 | 3.14 | U14 |
| 3.14 | 3.17 | Ethanolamine and isethionic acid |
| 3.17 | 3.19 | U15 |
| 3.19 | 3.22 | N-N-Nitrosodimethylamine |
| 3.22 | 3.24 | Carnitine |
| 3.24 | 3.25 | Carnosine |
| 3.25 | 3.26 | Taurine |
| 3.26 | 3.29 | TMAO |
| 3.29 | 3.32 | U16 |
| 3.36 | 3.366 | U17 |
| 3.366 | 3.37 | Theophylline |
| 3.38 | 3.395 | U18 |
| 3.395 | 3.405 | U19 |
| 3.41 | 3.42 | U20 |
| 3.45 | 3.456 | U21 |
| 3.456 | 3.464 | U22 |
| 3.468 | 3.476 | U23 |
| 3.476 | 3.49 | U24 |
| 3.52 | 3.526 | Caffeine |
| 3.535 | 3.545 | U25 |
| 3.545 | 3.565 | U26 |
| 3.57 | 3.58 | Glycine |
| 3.58 | 3.59 | U27 |
| 3.93 | 3.945 | Creatine phosphate |
| 4.095 | 4.115 | U28 |
| 4.16 | 4.21 | N-acetylglutamine |
| 4.215 | 4.24 | U29 |
| 4.34 | 4.355 | Tartrate |
| 4.502 | 4.51 | U30 |
| 4.51 | 4.53 | Ascorbate |
| 4.53 | 4.54 | U31 |
| 4.557 | 4.57 | U32 |
| 4.57 | 4.576 | U33 |
| 6.28 | 6.29 | U34 |
| 6.29 | 6.3 | U35 |
| 6.31 | 6.34 | U36 |
| 6.34 | 6.36 | U37 |
| 6.364 | 6.38 | U38 |
| 6.386 | 6.39 | U39 |
| 6.39 | 6.395 | U40 |
| 6.41 | 6.419 | U41 |
| 6.424 | 6.432 | Trans-aconitate |
| 6.425 | 6.433 | Urocanate |
| 6.44 | 6.46 | Chlorogenate |
| 6.473 | 6.48 | U42 |
| 6.48 | 6.494 | U43 |
| 6.495 | 6.51 | Fumarate |
| 6.52 | 6.535 | U44 |
| 6.538 | 6.544 | U45 |
| 6.555 | 6.575 | 2-furoate |
| 6.58 | 6.588 | U46 |
| 6.626 | 6.69 | 2-octenoate |
| 6.718 | 6.728 | Homovanillate |
| 6.755 | 6.78 | U47 |
| 6.78 | 6.795 | 2-hydroxyphenylacetate |
| 6.8557 | 6.88 | P cresol |
| 6.905 | 6.915 | 4-aminohippurate |
| 6.915 | 6.93 | 3-hydroxymandelate |
| 6.975 | 6.983 | Tyrosine |
| 7.01 | 7.02 | U48 |
| 7.05 | 7.065 | U49 |
| 7.065 | 7.08 | Histamines |
| 7.27 | 7.295 | U50 |
| 7.305 | 7.335 | U51 |
| 7.495 | 7.52 | Tryptophan |
| 7.53 | 7.59 | Hippurate |
| 7.67 | 7.687 | U52 |
| 7.687 | 7.72 | 3-inodoxyl sulfate |
| 8.026 | 8.034 | 3-methylxanthine |
| 8.46 | 8.47 | Formate |
| 8.64 | 8.655 | U53 |
| 8.67 | 8.69 | U54 |
| 8.77 | 8.8 | Pyrimidine |
| 8.798 | 8.81 | Nicotinurate/nicotinamide |
| 8.81 | 8.87 | Trigonelline |
| 8.88 | 8.91 | 1-methylnicotinamide |

Where multiple peaks were identified for a single metabolite, a representative peak with no/least spectral overlap was selected for inclusion in the analysis. Chemical shifts are displayed in parts per million (ppm) as the range between peak minima and maxima.

**Table S3:** Two pollutant models exploring the impact of co-exposure to combustion gases on metabolomic responses to airport particle exposure

| **Metabolite** | **Total PNC**  (5-95p= 120,280 #/ cm^3^) | |
| --- | --- | --- |
|  | **Accounting for NO_2_**  (5-95p = 33.2 µg/cm^3^) | **Accounting for CO**  (5-95p = 250 µg/cm^3^) |
|  | **Coef. (95% CI)** | **Coef. (95% CI)** |
| Taurine | **-0.300 (-0.569 - -0.031)** | **-0.296 (-0.546 - -0.046)** |
| Dimethylamine | **-0.027 (-0.044 – -0.009)** | **-0.023 (-0.040 - -0.007)** |
|  |  |  |
| **Metabolite** | **PNC < 20 nm**  (5-95p = 51,160 #/ cm^3^) | |
|  | **Accounting for NO_2_**  (5-95p = 33.2 µg/cm^3^) | **Accounting for CO**  (5-95p = 250 µg/cm^3^) |
|  | **Coef. (95% CI)** | **Coef. (95% CI)** |
| Taurine | **-0.293 (-0.547 - -0.039)** | **-0.319 (-0.577 - -0.060)** |
| Dimethylamine | **-0.025 (-0.042 - -0.008)** | **-0.023 (-0.040 - -0.006)** |

Data are presented as coefficients (coef.) of the relationship between exposure and Δ in metabolite concentration (post-pre) with 95% confidence intervals (CI) (expressed as the range between lower and upper values). All coefficients are adjusted for urinary ibuprofen and paracetamol markers, room temperature and humidity. Total PNC refers to particles smaller than 2.5µm in diameter, with a lower limit of 4nm, as measured by a condensation particle counter and SMPS. Numbers in bold represent significant relationships (p ≤ 0.05).

**Table S4:** Two-pollutant models for associations between Δ urinary metabolites and UFP produced through take-off and landing

| **Metabolite** | **Take-off PNC**  (5-95p= 56130 #/ cm^3^) | |
| --- | --- | --- |
|  | **Accounting for airport traffic PNC**  (5-95p= 5077 #/ cm^3^) | **Accounting for non-airport traffic PNC**  (5-95p= 15290 #/ cm^3^) |
|  | **Coef. (95% CI)** | **Coef. (95% CI)** |
| Taurine | -0.223 (-0.494 - 0.047) | -0.232 (-0.513 - 0.050) |
| Dimethylamine | **-0.019 (-0.037 - -0.001)** | **-0.020 (-0.038 - -0.001)** |
| Pyroglutamate | **-0.006 (-0.012 - -0.001)** | **-0.008 (-0.014 - -0.002)** |
| Isocitrate | 0.001 (-0.001 - 0.003) | **0.002 (> 0.000- 0.004)** |
| 2-hydroxyisobutyrate | -0.002 (-0.005 - < 0.000) | **-0.003 (-0.006 - < 0.000)** |
| **Metabolite** | **Landing PNC**  (5-95p = 31200 #/ cm^3^) | |
|  | **Accounting for airport traffic PNC**  (5-95p= 5077 #/ cm^3^) | **Accounting for non-airport traffic PNC**  (5-95p= 15290 #/ cm^3^) |
|  | **Coef. (95% CI)** | **Coef. (95% CI)** |
| Taurine | **-0.414 (-0.692 - -0.136)** | **-0.414 (-0.692 - -0.136)** |
| Dimethylamine | **-0.031 (-0.049 - -0.012)** | **-0.031 (-0.050 - -0.013)** |
| Pyroglutamate | **-0.002 (-0.004 - < 0.000)** | **-0.002 (-0.004 - < 0.000)** |
| Isocitrate | 0.001 (-0.001 - 0.004) | 0.002 (-0.001 - 0.004) |
| 2-hydroxyisobutyrate | -0.001 (-0.004 - 0.002) | -0.001 (-0.004 - 0.002) |

Data are presented as coefficients (coef.) of the relationship between exposure and Δ in metabolite concentration (post-pre) with 95% confidence intervals (CI). All coefficients are adjusted for urinary ibuprofen and paracetamol markers, room temperature and humidity. Numbers in bold represent significant relationships (p ≤ 0.05).
